# Supplementary material for: RGD-Coated Polymer Nanoworms for Enriching Cancer Stem Cells
Source: Cancers (Basel). 2022 Dec 30;15(1):234. doi: 10.3390/cancers15010234 (PMC9818073; doi:10.3390/cancers15010234)
Supplement: Supplementary file 1 [file cancers-15-00234-s001.zip › cancers-2063694-supplementary.pdf]

Table S1. Sphere size and theoretic calculation of average spherical cells/spheroid.

Sphere size (µm):	<100 (50-100)	100-200	200-300	300-400	>400 (400-600)
Average Size (µm):	~75	~150	~250	~350	~500
Radius r =	35	75	125	175	250
Cells/spheroid#	78	766	3544	9725	28353

#Notes: Spherical cells are slightly smaller than their parental HCT116 cells which have an average size of 18.4 µm in diameter (from Molecular Biology Handbook by ThermoFish Scientific) and thus assumed to have a diameter about 16.4 µm. Using the formular of sphere volume = $4/3\pi r^3$, we theoretically calculated the single spherical cell and spheroid volumes. The spherical cell numbers in each spheroid (Cells/spheroid) are then = spheroid volume/spherical cell volume.

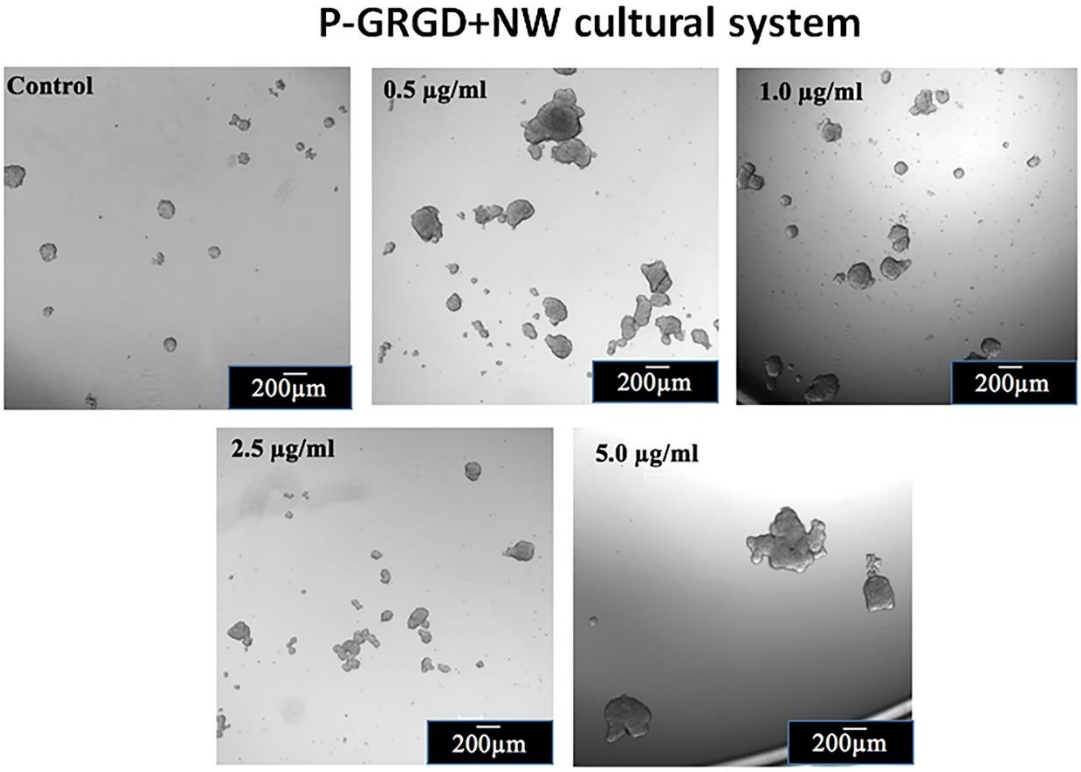

Figure S1. HCT116 spheroid morphology after sphere culture with/without P-GRGD+NW system. With the fixed nano-worm (NW) dose at 5 µg/mL and the different dose of P-GRGD (from 0.5 to 5.0 µg/mL), the system has different effects on sphere number and size. Control was the culture without any polymers.

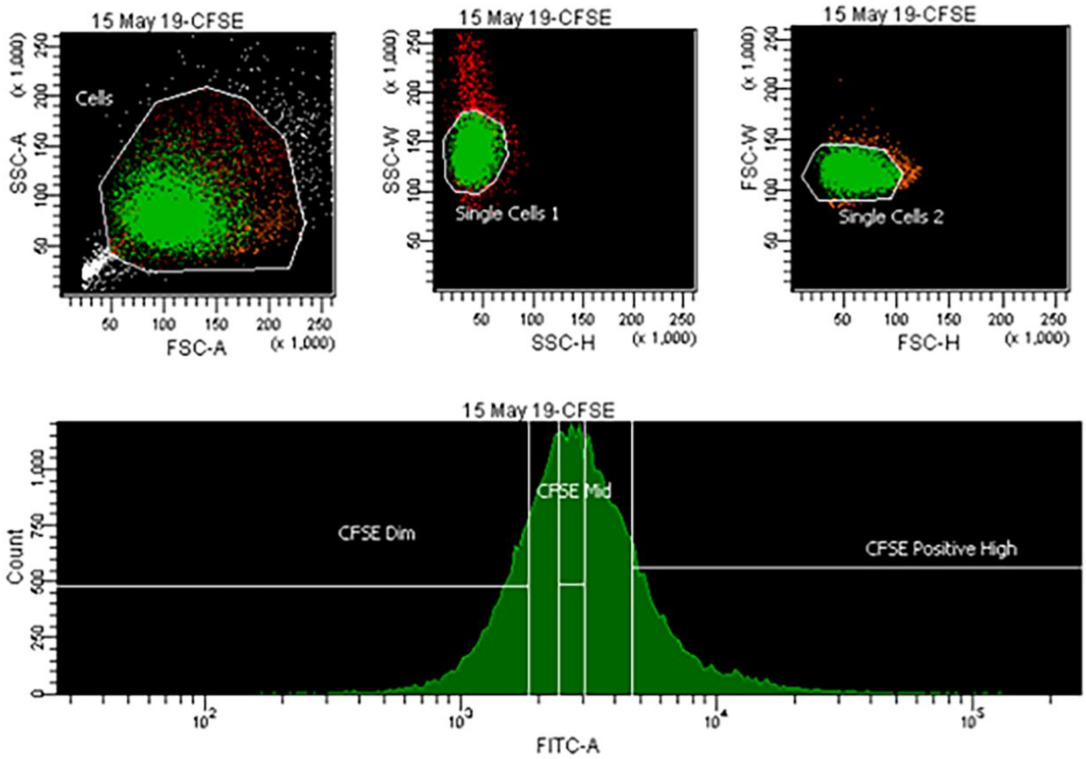

Figure S2. Fluorescence activated cell sorting analysis of CFSE-stained HCT116 cells. The HCT116 cells were stained with CFSE 3 days before flow cytometry sorting. The top panel shows the gating method: the top left excluded cell debris; the top middle and top right excluded doublets or cell clusters, selecting only single cells. The single cell population was sorted into 3 subpopulations according to their CFSE fluorescence intensity (Fast-dividing cells-CFSE Dim, median-CSFE-Mid, and slow-dividing cells-CFSE-Positive High, bottom panel).

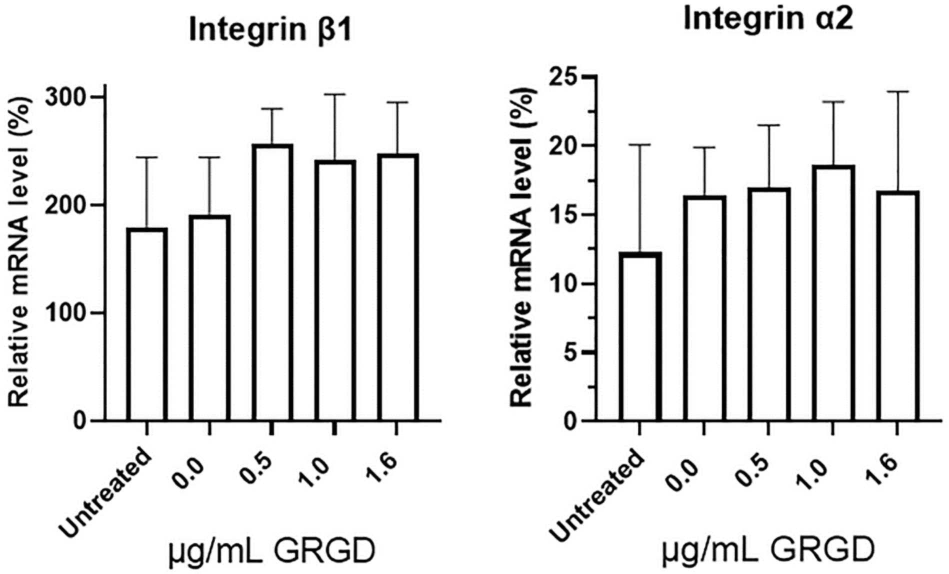

Figure S3. The real-time RT-PCR results of integrin receptor β1, and α2 after polymer-GRGD treatment. The relative expression was normalized by their house-keeping gene GAPDH expression. The group 0.0 indicates the polymer control.

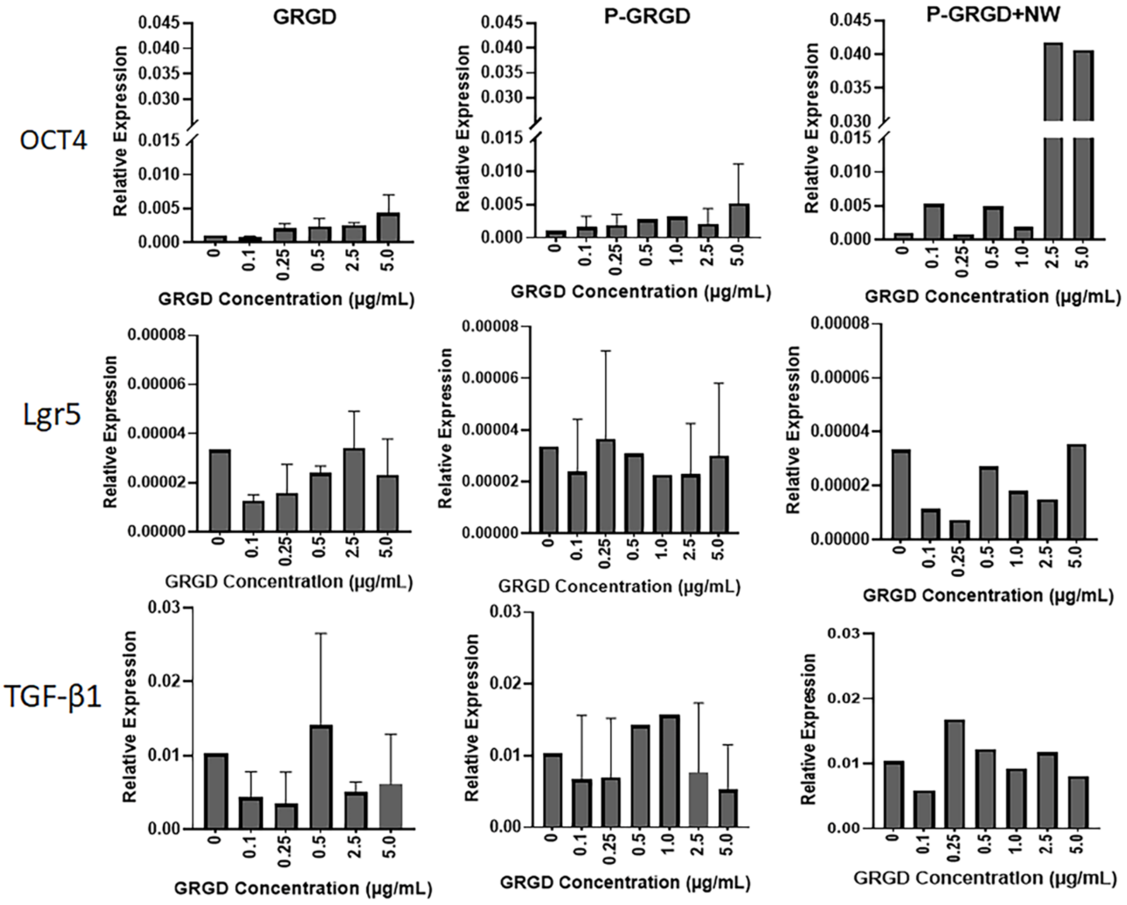

Figure S4. The effect of RGD and RGD-ECM on stem gene expression. Real-time RT-PCR results of some stem gene (including OCT4, Lgr5, and TGF-β1) expressions after different treatments with GRGD and polymeric GRDG at day 7 of sphere culture.
